# Prevalence of left ventricular systolic dysfunction and heart failure with reduced ejection fraction in men and women with type 2 diabetes mellitus: a systematic review and meta-analysis

**DOI:** 10.1186/s12933-018-0690-3

**Published:** 2018-04-18

**Authors:** Selma Bouthoorn, Aisha Gohar, Gideon Valstar, Hester M. den Ruijter, J. B. Reitsma, Arno W. Hoes, Frans H. Rutten, the Queen of Hearts Consortium, Hester M. den Ruijter, Frans H. Rutten, Arno W. Hoes, Gerard Pasterkamp, Marc Spaanderman, Chahinda Ghossein-Doha, Folkert Asselbergs, Erik Biessen, Michiel Bots, Caroline Cheng, Marijke Faas, Imo Hoefer, Johan Kuiper, Yigal Pinto, Torsten Plösch, Sicco Scherjon, Marianne Verhaar, Anton Jan van Zonneveld, Yolande Appelman, Rick Grobbee, Carolyn Lam, Tim Leiner

**Affiliations:** 1 Julius Center for Health Sciences and Primary Care, University Medical Center, Utrecht University, PO Box 85500, 3508 AB Utrecht, The Netherlands; 2 Laboratory for Experimental Cardiology, University Medical Center Utrecht, Utrecht University, Utrecht, The Netherlands

**Keywords:** LVSD, HFrEF, T2D, Sex, Prevalence, Meta-analysis

## Abstract

**Background:**

Type 2 diabetes mellitus (T2D) is associated with the development of left ventricular systolic dysfunction (LVSD) and heart failure with reduced ejection fraction (HFrEF). T2D patients with LVSD are at higher risk of mortality and morbidity than patients without LVSD, while progression of LVSD can be delayed or halted by the use of proven therapies. As estimates of the prevalence are scarce and vary considerably, the aim of this study was to retrieve summary estimates of the prevalence of LVSD/HFrEF in T2D and to see if there were any sex differences.

**Methods:**

A systematic search of Medline and Embase was performed to extract the prevalence of LVSD/HFrEF in T2D (17 studies, mean age 50.1 ± 6.3 to 71.5 ± 7.5), which were pooled using random-effects meta-analysis.

**Results:**

The pooled prevalence of LVSD was higher in hospital populations (13 studies, n = 5835, 18% [95% CI 17–19%]), than in the general population (4 studies, n = 1707, 2% [95% CI 2–3%]). Seven studies in total reported sex-stratified prevalence estimates (men: 7% [95% CI 5–8%] vs. women: 1.3% [95% CI 0.0.2.2%]). The prevalence of HFrEF was available in one general population study (5.8% [95% CI 3.7.6%], men: 6.8% vs. women: 3.0%).

**Conclusions:**

The summary prevalence of LVSD is higher among T2D patients from a hospital setting compared with from the general population, with a higher prevalence in men than in women in both settings. The prevalence of HFrEF among T2D in the population was only assessed in a single study and again was higher among men than women.

**Electronic supplementary material:**

The online version of this article (10.1186/s12933-018-0690-3) contains supplementary material, which is available to authorized users.

## Introduction

Type 2 diabetes mellitus (T2D) is a major risk factor for all types of heart failure (HF) and causes an increase in mortality and morbidity in patients with HF [1]. Under recognition of HF in T2D is an important problem with prevalence rates of unrecognized HF being reported as high as around 25% in the community aged 60 years and over [2]. T2D is commonly seen with coronary artery disease (CAD) [3]. Ischaemic heart disease is the usual cause of the left ventricular systolic dysfunction (LVSD) seen in HF with reduced ejection fraction (HFrEF). This subtype of HF is commonly reported as accounting for approximately 50% of all cases of HF, but this proportion may be lower in the general population/screen-detected HF [4] than in large HF cohorts including post-discharge/outpatient cohorts [5]. The number of people with T2D continues to rise worldwide having a profound impact upon society in terms of health burden and healthcare expenditure [6]. On the other hand, HFrEF is declining in prevalence, likely due to improved preventative and early treatment strategies, such as early revascularisation, which have led to a fall in the occurrence of ischaemic heart disease, notably acute ST-elevation myocardial infarction [7]. Despite this, the risk of all-cause mortality and cardiac hospitalization remains high and some studies report higher rates in HFrEF patients than in patients with heart failure with preserved ejection fraction (HFpEF) [8]. LVSD, the pre-clinical phase of HFrEF, is also associated with a poor outcome [9]. Unlike HFpEF, there is proven treatment for patients with LVSD that can delay or even prevent the progression of asymptomatic LVSD to symptomatic HF i.e. HFrEF [10]. Therefore identifying LVSD at an early pre-clinical stage is extremely useful in improving survival in T2D patients. Given the high prevalence of (unrecognized) HFrEF in T2D patients, the poor prognosis and available effective therapies, the implementation of screening-programmes in T2D patients with natriuretic peptides has been suggested to identify LV dysfunction in its pre-clinical phase [11]. However it is first imperative to know the exact prevalence rates of LVSD in T2D patients prior to implementing such approaches. Previous studies regarding prevalence rates of LVSD in T2D did not look at HFrEF and HFpEF separately, and also only looked at T2D patients in secondary care and not from the general population. Therefore we performed an extensive systematic review and meta-analysis, reviewing existing literature to estimate the prevalence of LVSD and HFrEF in T2D patients both in a hospital setting and a general population setting. Given the difference in prevalence rates of HF between men and women, and the higher prevalence of T2D in men, we were also interested to see if the prevalence rates differed by sex.

## Methods

### Literature search

A literature search was initially performed using the Medline and Embase databases including all studies up to and including May 2016. The search terms and synonyms used were ‘heart failure’, ‘systolic ventricular dysfunction’, ‘diabetes mellitus, type 2’, ‘prevalence’ and ‘incidence’. The search was repeated in March 2018 including all studies previously included and studies performed since the last literature search. For the exact search strategy see Additional file 1: Table S1. Of the studies retrieved for full text assessment, reference lists were screened for other relevant studies.

### Selection of articles

The following predefined inclusion criteria were applied: (i) The study reported the prevalence of HFrEF and/or LVSD in patients with T2D. (ii) The study population was derived from the population at large or from the hospital population. (iii) Only studies were included that used echocardiography to establish or confirm the diagnosis of HFrEF and/or LVSD. (iv) T2D defined by one of the following criteria: documentation in the medical record, physicians diagnosis, self-reported history, use of anti-diabetic agents and random serum glucose ≥ 200 mg/dL (or ≥ 11.1 mmol/L) or serum fasting glucose ≥ 126 mg/dL (or ≥ 7.0 mmol/L).

Only studies published in the English language were considered. Letters, editorials, case reports, practical guidelines and animal or in vitro studies were excluded.

If multiple studies were based on the same study population, the study with the largest population for data extraction was selected. Selection of publications and data extraction was done independently by three reviewers (SB, GV and AG). Consensus was used to resolve disagreement. If consensus could not be reached, a fourth reviewer (FR) was consulted.

### Quality assessment

A methodological quality assessment of each of the included studies was performed independently by three authors (SB, GV and AG). In case of discrepancies, consensus was reached after discussion. As there is no formal checklist available specifically designed to appraise risk of bias in prevalence studies, we based our assessment on the risk of bias tool of Hoy et al. [12]. This is a new risk of bias tool for prevalence studies based on a modification of an existing tool and on the approach of the QUADAS-2 (tool for the quality assessment of diagnostic accuracy studies) [13]. Signalling questions were used to identify potential problems in the design, conduct and analysis of a study that might introduce bias or raise concerns about the applicability of the findings. The following signalling questions were used:

- Has the correct population/setting been targeted in order to answer the research question (T2D patients in the general population, referral centres, hospital centre)?
- Is the sampling frame a true or close representation of this target population intended by the research question?
- Is an unselected (random/consecutive) sample of patients invited to participate?
- Is the response rate ≥ 75% or did a non-response analysis show no difference between participants and nonparticipants?
- Is an acceptable case definition for LVSD and/or HFrEF used in the study?
- Is the instrument to measure LVSD and/or HFrEF valid?
- Is the same mode of data collection used for all subjects?
- Is it unlikely that the handling of missing (endpoint) data introduced bias?
- Were the numerator(s) and denominator(s) for the parameter of interest appropriate?

All signalling questions were scored with either low or high risk of bias. Overall risk of bias was classified as low (if ≤ 1 question was answered high), medium (if 2–3 questions were answered high) or high (if > 3 questions were answered high).

### Data extraction and analysis

Information on study characteristics was collected with a data extraction form and comprised of first author’s name, publication year, source population and setting, age, number of participants, duration of T2D, exclusion criteria, echocardiographic measurements used, left ventricular ejection fraction (LVEF) threshold used and prevalence estimates of HFrEF and/or LVSD. Prevalence numerators and denominators were extracted from the studies. Individual study prevalence and corresponding 95% confidence intervals (95% CI) were calculated for all the included studies. To perform the meta-analysis, the prevalence data were log transformed so that the data followed a normal distribution. Given the inclusion of some studies with a zero prevalence of LVSD or HFrEF, the Freeman–Tukey transformation was performed [14]. A random-effects model was used to obtain pooled estimates (with corresponding 95% CI) of the transformed prevalence data. This model takes into account the between-study heterogeneity. Heterogeneity was assessed using the Cochrane Q test and the I^2^ statistic [15]. The pooled prevalence estimate was calculated for all of the included studies, and separately for studies concerning the general population and hospital population. Sex-specific pooled estimates were calculated for both sexes with the two settings combined. Results of the meta-analysis are presented as Forest plots showing prevalence proportions with corresponding 95% CIs for each study and the overall random-effects pooled estimate. Publication bias was first assessed by visually inspecting the distribution of observed studies on a funnel plot (Additional files 2: Figure S1 and Additional file 3: Figure S2). To quantify the degree of bias illustrated in the funnel plot, the Begg’s rank correlation test and Egger’s linear regression were used [16, 17]. A *p* < 0.05 was considered statistically significant. All statistical analyses were performed in R by using the ‘metafor’ package [18].

## Results

### Search results and characteristics

**Fig. 1:**
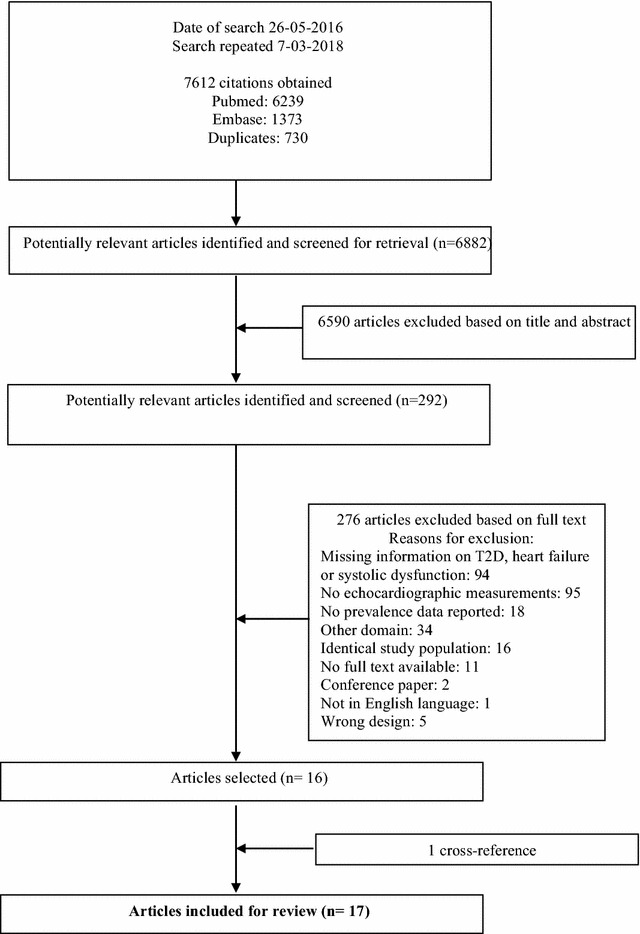
Flow chart of the process for selection of relevant articles

In total the search resulted in 6882 potentially relevant studies. These studies were first screened on title and then on abstract for eligibility. Full text articles were additionally screened of 292 studies for more detailed information. The main reasons for exclusion included the lack of T2D in the population, no information regarding HF or LVSD/HFrEF and lack of echocardiographic data. Thus 17 studies were eventually included in this review. Details of the selection process are provided in Fig. 1. Study characteristics and quality assessment of all the included studies are shown in Table 1. Of the 17 included studies, 13 included participants derived from a hospital setting [3, 19–30]. The majority of these hospital setting studies were in the outpatient setting with only one including hospitalized patients. Four studies consisted of patients from the general population [2, 31–33]. All studies consisted of data regarding the prevalence of LVSD with only one study containing data on the prevalence of HFrEF in addition to LVSD (Table 1) [2]. The mean age in the studies ranged from 50.1 ± 6.3 to 71.5 ± 7.5. The LVEF cut-off point ranged from 45–55% with the majority of studies using 50% (n = 12). Most studies had a medium risk of bias (n = 12).

**Table 1:** General characteristics and quality assessment of included studies

Author (year)	Source population and setting	Age^a^	Participants (% male)	T2D duration (years) [mean ± SD or median (range)]	Exclusion criteria	Cut-point LVEF to separate LVSD from LVDD (%)	Presence of heart failure assessed (yes/no)	Risk of bias (low/high)									Overall risk (low/medium/high)
								a	b	c	d	e	f	g	h	i	
Annonu (2001)	Patients attending the Diabetic Center of Cairo University hospital, Egypt	39–64 57 ± 6.8	66 (53%)	Not reported	Insulin use, alcoholism, clinical or electrocardiographic evidence of heart diseases and hypertension	50	No	L	H	H	H	L	L	L	L	L	Medium
Fang (2005)	Asymptomatic patients from the ambulatory Diabetes Clinic at Princess Alexandra Hospital, Australia.	No age range or overall mean age reported	101 (not reported)	Not reported	History of complaints of cardiac disease, history of coronary artery disease, valvular disease, atrial fibrillation, severe arrhythmias and congenital heart disease	50	No	L	H	L	H	H	L	L	H	H	High
Dawson (2005)	Random volunteers from the Diabetes Centre, Ninewells Hospital, Scotland	63.8 ± 10.6	500 (61.6%)	6.0 ± 5.5	Frailty and inability to give written informed consent	45	No	L	L	L	L	L	L	L	H	H	Medium
Albertini (2008)	Consecutive asymptomatic patients admitted at the Avicenne Hospital endocrinology unit, France	59.8 ± 1.5	91 (54%)	13 ± 1.1	Previous or suspected history of heart disease, intrinsic lung or overt renal disease, incomplete echocardiographic data or poor echogenicity	50	No	L	H	L	H	L	L	L	L	L	Medium
Chaowalit (2006)	Patients referred for clinically indicated dobutamine stress echo, US	67 ± 11	2349 (57%)	Not reported	None	55	No	H	H	H	L	L	L	L	L	L	Medium
Srivastava (2008)	Patients referred for echocardiography as part of a routine complications surveillance programme, mainly by general practitioners (80%) and 20% from the hospital, at the Diabetic Clinic at Austin Health, Australia	62 ± 1	229 (58%)	10 ± 1	None	50	No	L	L	H	H	L	L	L	H	L	Medium
Poulsen (2010)	Patients referred, for the first time, for diabetes education or poorly regulated diabetes to the Diabetes Clinic at Odense University Hospital, Denmark	58.6 ± 11.3	305 (54%)	4.5 ± 5.3	History of CVD, malignancy or End-stage kidney disease, pregnancy, body weight > 150 kg, physical or mental disability, not able to provide inform consent	50	No	L	H	L	H	L	L	L	L	L	Medium
Aigbe (2012)	Randomly selected patients at the University Teaching Hospital, Nigeria	26–80 55.4 ± 11.6	300 (150 cases, 43% male)	4.5 ± 4.5	Hypertension, pregnancy, sickle cell disease and structural heart disease	50	No	L	H	L	H	L	L	L	L	H	Medium
Boonman-de Winter (2012)	Patients enrolled in the Diabetes Care programme of the Center for Diagnostic Support in Primary Care, the Netherlands	71.5 ± 7.5	581 (53%)	Not reported	None	45	Yes	L	L	L	H	L	L	L	L	L	Low
Cioffi (2012)	Non-institutionalized subjects > 45 years of age participating in the Dysfunction in DiAbetes’ (DYDA) study recruited in 37 diabetes referral centres, Italy	61 ± 7	751 (61%)	7 (3–13)	Myocardial infarction, myocarditis, HF, coronary heart disease, alcoholic cardiomyopathy, primary hypertrophic cardiomyopathy, asymptomatic known LVD, prior myocardial revascularization, valvular heart disease, atrial fibrillation, electrocardiographic findings of myocardial ischaemia, DMI and severe systematic disease with life expectancy < 2 years	50	No	L	H	L	L	L	L	L	L	L	Low
Faden (2013)	Consecutive non-institutionalized subjects > 18 years of age attending a prospective, multicentre study, (SHORTWAVE) in cardiology and diabetes referral centres in 4 hospitals, Italy	69 ± 10	386 (57%)	5 (2–10)	Myocardial infarction, dilated cardiomyopathy or HF, primary hypertrophic cardiomyopathy, prior myocardial revascularization, valvular disease, atrial fibrillation, chronic pulmonary disease, DMI	Not reported	No	L	H	L	H	L	L	L	L	L	Medium
Dodiyi-Manuel (2013)	Patients attending the Medical Outpatient Department of the University of Port Harcourt Teaching Hospital, Nigeria	36–65 50.8 ± 9.1	180 (90 DMII patients, 43% male)	3.4 ± 2.9	Hypertension (> 140/90 mm Hg), anti-hypertensive medications, valvular abnormalities and wall motion abnormalities	55	No	L	H	H	H	L	L	L	L	L	Medium
Chen (2014)	Consecutive patients treated with stable hypoglycaemic medication for at least 3 months recruited from the medical outpatient clinic of Queen Mary Hospital, Hong Kong, China	62 ± 9	95 (39%)	10 ± 8	History or clinical symptoms of cardiovascular disease, including CAD, MI, stroke or peripheral vascular disease, renal impairment (eGFR < 30 mL/min/1.73 m^2^), liver failure, SLE, rheumatoid arthritis, systemic sclerosis	50	No	L	H	L	H	L	L	L	L	L	Medium
Dandamundi (2014)	Random sample of residents participating in the Rochester Epidemiology Project, Olmsted County, USA	Normal LV function: 62.6 ± 9.1	2042 (136 DMII patients, 60% male)	Not reported	Missings on systolic or diastolic assessments	50	No	L	L	L	H	L	L	L	L	L	Low
		Diabetic cardiomyopathy: 68.5 ± 10.6															
		Any LV dysfunction: 67.6 ± 9.2															
Chaudhary (2015)	Normotensive patients with newly diagnosed (within 1 month) DMII recruited from the SVBP Hospital, LLRM Medical College, Meerut, India	30–60 50.1 ± 6.3	100 (65%)	New onset	Hypertension > 130/80, abnormal ECG, already diagnosed DMII, antidiabetic treatment, valvular heart disease, ischaemic and hypertensive heart disease, congestive HF, cardiomyopathie, renal failure, COPD, severe anemia and haemoglobinopathies	50	No	L	H	H	H	L	L	L	L	L	Medium
Xanthakis (2015)	Population based longitudinal study with DM or metabolic syndrome	59.1 ± 10.46	761 (31%)	Not reported	History of CVD	50	No	L	L	L	H	L	L	L	L	L	Low
Jørgensen (2016)	Patients with DMII recruited from Sterno Diabetes Centre and the Centre for Diabetes research in Copenhagen	65.5 (58.8, 71.4)	1030 (65.9%)	11 (5.5, 17)	None	50	No	L	L	H	H	L	L	L	L	L	Medium

^a^Values indicate the age range, mean ± standard deviation or median (range)

### Prevalence of LVSD and HFrEF

**Fig. 2:**
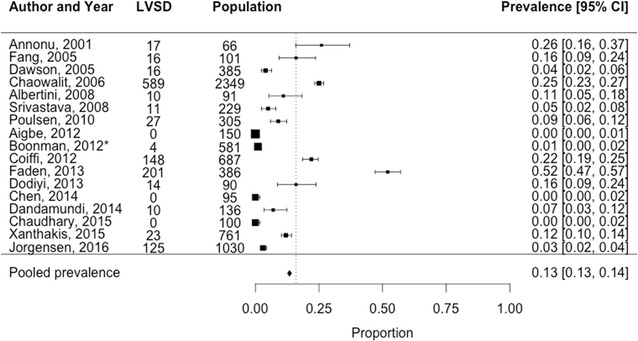
Prevalence of left ventricular systolic dysfunction among 7542 T2D patients in both the general and hospital population. *Study by Boonman et al. is a HF-screening study which was performed in the general population. The corresponding estimate of LVSD is made up from a sample excluding individuals with previously known HF at the start of the study

**Fig. 3:**
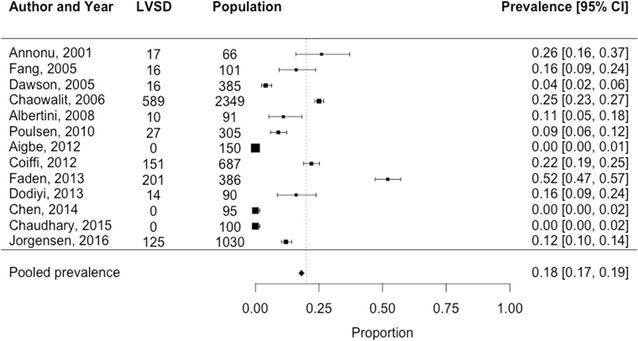
Prevalence of left ventricular systolic dysfunction among 5835 T2D patients in the hospital setting

**Fig. 4:**
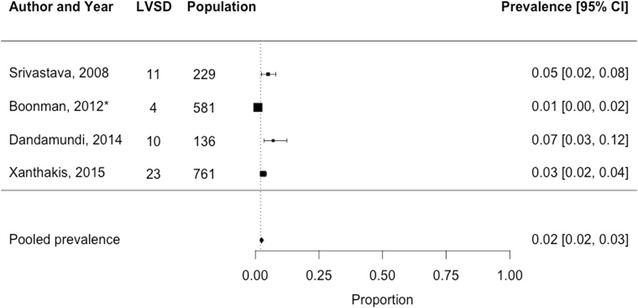
Prevalence of left ventricular systolic dysfunction among 1707 T2D patients in the general population setting. *Study by Boonman et al. is a HF-screening study which was performed in the general population. The corresponding estimate of LVSD is made up from a sample excluding individuals with previously known HF at the start of the study

The pooled prevalence estimate for all 17 included studies (both hospital setting and general population setting) was 13% [95% CI 13–14%] (Fig. 2). For the 13 studies in the hospital population and the 4 studies in the general population the pooled estimates were 18% [95% CI 17–19%] and 2% [95% CI 2–3%] respectively (Figs. 3 and 4). Estimates ranged from 0 to 52% in the hospital setting and 1 to 7% in the general population setting. Heterogeneity was higher for the hospital setting (Q = 593.3, p < 0.001, I^2^ = 98.5% than the general population setting (Q = 24.8, p < 0.001, I^2^ = %). There was no potential risk of publication bias Begg’s (p = 0.95 and p = 0.33 respectively) and Egger’s test (p = 0.31 and p = 0.06 respectively).

Sex-stratified prevalence rates were only available for seven studies (both hospital setting and general setting combined). In three of these studies the total prevalence of LVSD was 0%; thus the overall prevalence in the seven studies reporting sex-specific findings was considerably lower than in the 17 studies combined. Sex-specific pooled estimates in these seven studies were 7% [95% CI 5–8%] for men and 0.1% [95% CI 0.6–2%] in women. The sex-specific pooled estimates from the hospital setting were: 9% [95% CI 7–10%] in men vs. 2% [95% CI 1–4%] in women. Only one study looked at sex-specific prevalence estimates from the general population (1.3% [95% CI 0–3%] in men vs. 0.0% [95% CI 0–0.1%] in women).

**Fig. 5:**
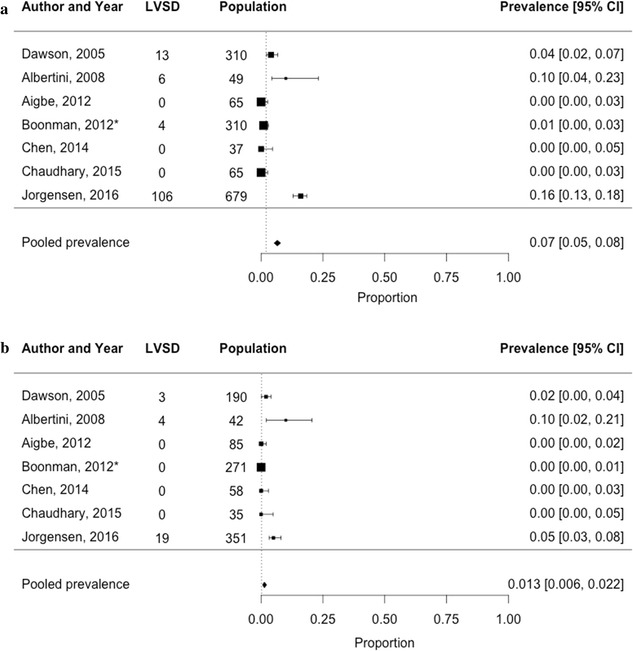
Prevalence of left ventricular systolic dysfunction among **a** 1515 male T2D patients and **b** 1032 female T2D patients in both the general and hospital population. *Study by Boonman et al. is a HF-screening study which was performed in the general population. The corresponding estimate of LVSD is made up from a sample excluding individuals with previously known HF at the start of the study

The prevalence of HFrEF was only available in one study, performed by Boonman et al. using a sample from the general population of T2D patients aged 60 years or over. As this study screened for previously unknown HFrEF in addition to LVSD, individuals with an established diagnosis of HF were excluded from the main analyses from their study. The estimates of LVSD of participants without previously known HF at the start of the study can be viewed in the forest plots (Figs. 2 and 4) and stratified by sex in Fig. 5. In this study, Boonman et al. reported the prevalence of HFrEF, based on an LVEF < 45% and including individuals known to have HF at the start of the study to be 5.8% [95% CI 3.9–7.6%]. The corresponding prevalence of HFrEF in men and women without previously known HF was higher in men than women (6.8% vs. 3.0%).

## Discussion

Our systematic review and meta-analysis showed that the prevalence of LVSD is on average higher when specifically looking at studies enrolling patients from a hospital setting (18% [95% CI 17–19%] than those from the general population (2% [95% CI 2–3%]. The latter includes the correct denominator when assessing the prevalence in T2D, and as such provides the more accurate prevalence estimate [34]. In only six hospital studies with in total 1205 men and 761 women, sex-specific estimates were reported; the prevalence of LVSD among men with T2D was 9% [95% CI 7–10%] and in women 2% [95% CI 1–4%]. In the only study from the general population reporting sex-specific estimates the prevalence of LVSD in men was 1.3% [95% CI 0–3%] and in women 0% [95% CI 0–0.1%]. There was large heterogeneity in the published prevalences and the population making up the hospital setting was more heterogeneous than the population included in the general population studies. It is important to note that heterogeneity is generally higher in diagnostic/prevalence studies than drug trials as these studies consist of ‘real life’ people from the population with a multitude of comorbidities and of an older age. This is in contrast to participants in drug trials who are subject to strict inclusion and exclusion criteria and therefore are generally younger and usually have only one disease, which leads to lower heterogeneity. The reasons for the heterogeneity we see in our review include the selection criteria used. Although, T2D patients in the hospital setting are more diseased and have more comorbidities than those from the general population, in the studies providing estimates, 10 out of the 13 hospital population studies excluded patients with a history of cardiovascular disease [19, 20, 22, 23, 25, 27, 33] or other diseases, such as hypertension [19, 24, 26, 30], atrial fibrillation [20, 25, 33] and renal impairment [23, 27], that are (potentially) in the causal pathway in the development of LVSD/HFrEF. This will have resulted in an underestimation of the prevalence. One reason for exclusion of these diseases provided by the authors of such publications was the independent impact these diseases have on LV function [27]. This, however, is somewhat counterintuitive as HFrEF does not merely develop ‘out of the blue’, that is, in patients without any CV history, known or unknown. Another reason for the possible heterogeneity in the hospital population studies could be the duration of T2D. This was not reported in 4 out of the 13 studies. However there does not appear to be a relationship between the duration of T2D and the prevalence of LVSD. Age may explain some of the heterogeneity present as the studies with the oldest participants (Chaowalit et al. and Faden et al.) also had the highest reported prevalence of LVSD. The study with the youngest participants (Chaudhary et al.) had a LVSD prevalence of 0%.

There were only four studies performed in a sample from the general population. Three of these studies (Srivastava et al., Dandamundi et al. and Xanthakis et al.) showed similar estimates, using LVEF 50% as a cut point and only providing data on LVSD. The fourth study, by Boonman et al. used LVEF 45% as a cut point and it was the only study providing data on HFrEF in addition to LVSD. This study presented an estimate of 1% for LVSD, while for HFrEF it was 5.8%. It is important to note that this study was a HF-screening study with the aim of identifying previously unrecognized HF in the community. Therefore participants with previously known HF were removed from the study and from analyses involving LVSD. The authors did provide data on the estimate of HFrEF including participants previously known to have HF. Given the nature of the study, participants with a LVEF < 45% were scrutinized for the slightest suggestion of symptoms, such as shortness of breath (MRC 2 was considered to be dyspnoea) and were subsequently labelled as HFrEF instead of LVSD. It is only the symptoms, (and possibly signs) of HF that may be considered to be the difference between LVSD and HFrEF, which is a clinician-based observation. This may explain the somewhat lower estimate of LVSD seen between this study (1%) compared to the other general population studies by Srivastava et al. (5%), Dandamundi et al. (7%) and Xanthakis et al. (3%) which did not assess HF symptoms and thus included “symptomatic” LVSD (i.e. HFrEF) as the numerator.

CAD can be silent in patients with T2D, more so than in those without T2D. Therefore it could be more difficult to pick up HF in these patients in the pre-clinical phase. In addition, due to the non-specific nature of the disease, HF can be difficult to diagnose prior to echocardiography, therefore remaining unrecognized in the community, leaving patients untreated. We report an overall higher prevalence of LVSD in the T2D population compared with the prevalence of LVSD in the general population [35]. Given that there is proven therapy available for LVSD/HFrEF [10], our results highlight the importance of timely detection of HF in men and women with T2D. Screening by way of measuring NTproBNP levels to identify patients early is a possible option so that management can be provided in a timely manner. Patients with both HFrEF and T2D have been found to have high mortality rates, with up to a tenfold increase in mortality [1]. This has been explained by the association of T2D with features of adverse structural and functional cardiac remodelling in patients with HF [36]. This high mortality, in addition with the rapidly rising prevalence of T2D globally due to obesity and lack of exercise [6], only strengthens the argument for screening of HF in these patients. New, more sensitive echocardiographic techniques have enabled the non-invasive detection of LVSD underlying HF in diabetes at an early stage [37] making it easier to diagnose LVSD and HF in a high risk population, such as T2D as we see here. The cost-effectiveness of screening for HF in diabetic patients has also already been proven [38] further highlighting the usefulness of screening high-risk patient groups, such as T2D for HF.

Men are known to be at a higher risk of developing LVSD/HFrEF than women. This is likely due to the higher prevalence of coronary macrovascular disease seen in men than women. Of the seven studies with sex-stratified data, only the study by Boonman et al. was performed in the general population. The overall prevalence of HFrEF was indeed higher in men than in women.

There are a number of limitations of our review. As mentioned, there was significant heterogeneity between the hospital-based studies. This, however is a known feature of meta-analyses regarding prevalence rates [39]. In addition to the exclusion criteria used, this can also be explained, albeit to a lesser extent, by the differences in cut-off LVEFs used for each study with cut-offs ranging from 45 to 55%, the applied cut points for other echocardiographic parameters, and also by differences in age, year of study and sample size. Low numbers of included studies set in the general population is also a limitation of this review as we were unable to adequately compare the pooled prevalence of these patients with the pooled prevalence of the patients included within the hospital setting. The same also holds true when comparing the pooled prevalence rates between men and women.

## Conclusion

In conclusion, from 17 studies including a low number of patients in total, the summary prevalence of LVSD among T2D patients in a hospital setting is much higher (around 18%) than in samples from the general population (around 2%). The prevalence is higher in men as compared to women in both settings. The prevalence of HFrEF, only assessed in one study, was also higher among men than women.

## Additional files

**Additional file 1: Table S1.** Search terms used in Embase and Medline.

**Additional file 2: Figure S1.** Funnel plot of studies measuring LVSD among T2D patients from a hospital setting.

**Additional file 3: Figure S2.** Funnel plot of studies measuring LVSD among T2D patients from the general population.
