# Supplementary material for: Prevalence of left ventricular systolic dysfunction and heart failure with reduced ejection fraction in men and women with type 2 diabetes mellitus: a systematic review and meta-analysis
Source: Cardiovasc Diabetol. 2018 Apr 18;17:58. doi: 10.1186/s12933-018-0690-3 (PMC5907399; doi:10.1186/s12933-018-0690-3)
Supplement: Supplementary file 1 — Additional file 1: Table S1. Search terms used in Embase and Medline. [file 12933_2018_690_MOESM1_ESM.docx]

**Additional file**

**Table S1. Search terms used in Embase and Medline**

| **Embase** |
| --- |
| **'heart failure'**/exp OR (**'heart failure'**:ab,ti AND (**'diastolic'**:ab,ti OR **'systolic'**:ab,ti)) OR ‘ejection fraction’:ab,ti OR **'heart ventricle function'**/exp OR (**'failure'**:ab,ti OR **'decompensation'**:ab,ti OR **'insufficiency'**:ab,ti OR **'dysfunction'**:ab,ti OR **'disfunction'**:ab,ti AND (**'ventricular'**:ab,ti OR **'cardiac'**:ab,ti OR **'heart'**:ab,ti OR **'myocardial'**:ab,ti)) AND (**'non insulin dependent diabetes mellitus'**/exp OR **'diabetes mellitus'**:ab,ti OR **'t2d'**:ab,ti) AND (**'prevalence'**/exp OR **'prevalence'**:ab,ti OR **'incidence'**/exp OR **'incidence'**:ab,ti OR **'occurrence'**:ab,ti OR **'frequency'**:ab,ti OR **'rate'**:ab,ti OR **'rates'**:ab,ti OR **'frequencies'**:ab,ti OR **'percentage'**:ab,ti OR **'percentages'**:ab,ti OR **'hf ref'**:ab,ti OR **'hf pef'**:ab,ti) AND [embase]/lim NOT [medline]/lim AND ([dutch]/lim OR [english]/lim) AND (**'article'**/it OR **'article in press'**/it OR **'review'**/it) |
| **Medline** |
| (((((((("Heart Failure"[Mesh:noexp]) OR ((heart failure[Title/Abstract]) AND ((diastolic[Title/Abstract]) OR systolic[Title/Abstract]))) OR ejection fraction[Title/Abstract] OR "Ventricular Dysfunction"[Mesh]) OR (((((((failure[Title/Abstract]) OR decompensation[Title/Abstract]) OR insufficiency[Title/Abstract]) OR dysfunction[Title/Abstract]) OR disfunction[Title/Abstract])) AND (((ventricular[Title/Abstract]) OR cardiac[Title/Abstract]) OR heart[Title/Abstract] OR myocardial[Title/Abstract])))) AND ((("Diabetes Mellitus, Type 2"[Mesh:noexp]) OR diabetes mellitus[Title/Abstract]) OR T2D [Title/Abstract]))) AND (((("Prevalence"[Mesh]) OR prevalence[Title/Abstract]) OR "Incidence"[Mesh]) OR ((((((((incidence[Title/Abstract]) OR occurence[Title/Abstract]) OR frequency[Title/Abstract]) OR rate[Title/Abstract]) OR rates[Title/Abstract]) OR frequencies[Title/Abstract]) OR percentage[Title/Abstract]) OR percentages[Title/Abstract] OR (Hf ref[Title/Abstract]) OR (Hf pef[Title/Abstract])))) |
