# Supplementary figures and images for: Prevalence of left ventricular systolic dysfunction and heart failure with reduced ejection fraction in men and women with type 2 diabetes mellitus: a systematic review and meta-analysis
Source: Cardiovasc Diabetol. 2018 Apr 18;17:58. doi: 10.1186/s12933-018-0690-3 (PMC5907399; doi:10.1186/s12933-018-0690-3)

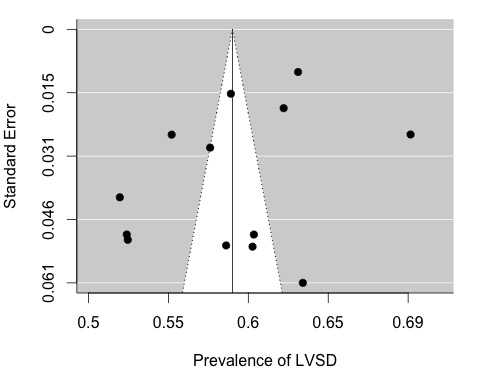

Supplement: Supplementary file 2 — Additional file 2: Figure S1. Funnel plot of studies measuring LVSD among T2D patients from a hospital setting. [file 12933_2018_690_MOESM2_ESM.jpeg]

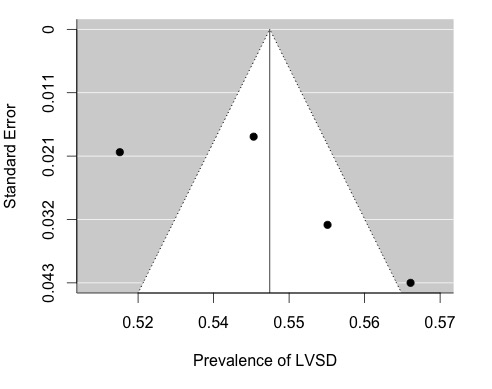

Supplement: Supplementary file 3 — Additional file 3: Figure S2. Funnel plot of studies measuring LVSD among T2D patients from the general population. [file 12933_2018_690_MOESM3_ESM.jpeg]
